# Label-Free Kinetic Studies of Hemostasis-Related Biomarkers Including D-Dimer Using Autologous Serum Transfusion

**DOI:** 10.1371/journal.pone.0145012

**Published:** 2015-12-14

**Authors:** Heiko Rühl, Christina Berens, Anna Winterhagen, Jens Müller, Johannes Oldenburg, Bernd Pötzsch

**Affiliations:** Institute of Experimental Hematology and Transfusion Medicine, University Hospital Bonn, Bonn, Germany; Maastricht University Medical Center, NETHERLANDS

## Abstract

The objective of this study was to evaluate the elimination kinetics of hemostasis-related biomarkers including the prothrombin activation fragment F1+2, thrombin-antithrombin complex (TAT), plasmin-α_2_-antiplasmin complex (PAP), and D-dimer in humans. Autologous serum was used as a biomarker source and infused into 15 healthy volunteers. Serum was prepared from whole blood in the presence of recombinant tissue-type plasminogen activator (final concentration 20 μg/mL) to induce plasmin generation required for PAP and D-dimer formation. Serum transfusions (50 mL/30 min) were well tolerated by all subjects. Endogenous thrombin formation was not induced by serum infusions as measured using a highly sensitive oligonucleotide-based enzyme capture assay. Median peak levels (x-fold increase over baseline) of F1+2, TAT, PAP, and D-dimer of 3.7 nmol/L (28.9), 393 ng/mL (189.6), 3,829 ng/mL (7.0), and 13.4 mg/L (34.2) were achieved at the end of serum infusions. During a 48 h lasting follow-up period all biomarkers showed elimination kinetics of a two-compartment model. Median (interquartile range) terminal half-lives were 1.9 (1.3–3.6) h for F1+2, 0.7 (0.7–2.6) h for TAT, and 10.8 (8.8–11.4) h for PAP. With 15.8 (13.1–23.1) h the D-dimer half-life was about twice as long as previously estimated from radiolabeling studies in animals and small numbers of human subjects. The serum approach presented here allows label-free and simultaneous analysis of the elimination kinetics of various hemostasis-related biomarkers. Based on these data changes in biomarker levels could more precisely used to estimate the activity level of the hemostatic system.

## Introduction

Hemostasis-related biomarkers are specific products that are generated during activation and regulation of the clotting process and are released into the circulating blood. Established markers include the prothrombin activation fragment F1+2 [1,2], thrombin-antithrombin complex (TAT) [2,3], plasmin-α_2_-antiplasmin complex (PAP) [4], and D-dimer [5,6]. F1+2 is a measure of the amount of thrombin formed, while TAT and PAP are measures of the amount of thrombin and plasmin inhibited by their corresponding endogenous inhibitors [7,8]. D-dimer is a degradation product of cross-linked fibrin that is formed by the sequential processes of clot formation and fibrinolysis and is therefore a compound measure of thrombin-catalyzed fibrin formation and subsequent plasmin-catalyzed fibrin degradation [6,7]. The most notable clinical application of D-dimer measurement is in the diagnosis of venous thromboembolism (VTE), where D-dimer levels below the threshold reach negative predictive values of above 90% [9,10].

Changes in plasma levels of these biomarkers can be used to estimate the rates of thrombin and fibrin formation, and activation of the fibrinolytic system in healthy individuals and in various clinical conditions [7,11,12]. In addition, measurement of F1+2 and TAT might improve the specificity of D-dimer testing in diagnosing VTE, and biomarker-guided anticoagulant treatment might form the basis of a personalized anticoagulant strategy [13–16]. Since biomarkers are being formed and cleared simultaneously, information on their half-lives is of particular importance when they will be used in vivo to estimate the level of hemostasis activation. Currently, the knowledge about their elimination kinetics is limited. Based on studies on the plasma disappearance of radiolabeled fibrinogen or fibrin fragments, D-dimer is believed to be cleared from the circulation with a half-life of 9–10 h [17–23]. However, studies in humans are sparse, and they have been conducted with small numbers of subjects only [21,23]. This also applies to studies on the elimination kinetics of F1+2 [24], TAT [25–27], and PAP [28], in which similar radiolabeling techniques were used.

In the study presented here, a different approach was followed by using serum of healthy human probands as source of hemostasis-related biomarkers. The concentration of D-dimer in serum is comparable to that in plasma since fibrinolysis is not activated in the absence of endothelial cells [29]. Therefore recombinant tissue-type plasminogen activator (rt-PA) was used to induce plasmin formation required for PAP and D-dimer formation. After transfusion of this autologous serum high enough plasma levels of hemostasis-related biomarkers were reached to study their elimination kinetics over time. This approach has several advantages over the radiolabeling approach: (1) The parameters of interest can be measured directly, and do not require radioactivity measurement. (2) It allows simultaneous assessment of the elimination of several biomarkers, which would require the use of different isotopes in the radioactive labeling technique. (3) As there is no radiation exposure of the probands, the administration of higher amounts of biomarkers in humans is possible. By doing so, plasma levels can be achieved that are similar to those present in clinical situations of coagulation activation.

Transfusion of serum can activate the coagulation cascade [30]. To detect a serum-induced activation of the clotting cascade, plasma levels of free thrombin were monitored using a highly sensitive oligonucleotide-based enzyme capture assay (OECA) that has been proven to detect surgery-induced thrombin formation in a previous study [31].

## Patients, Materials and Methods

This prospective study was conducted from July 2014 to February 2015 at the Institute of Experimental Hematology and Transfusion Medicine, Bonn, Germany. The study proposal was approved by the Institutional Review Board and Ethics committee of the University Hospital of Bonn. Written informed consent was obtained in compliance with the declaration of Helsinki.

### Volunteer selection

Healthy volunteers 18–60 years of age with ≥ 50 kg body weight and adequate peripheral vein status were eligible for inclusion. Exclusion criteria consisted of blood donation within the preceding two months, a history of cardiovascular or malignant diseases, acute infections, anemia, impaired hepatic or renal function tests (transaminases, γ-glutamyl transferase, urea, and creatinine in serum). For safety reasons individuals with abnormalities in a thrombophilia screen, that consisted of testing for activity levels of antithrombin (AT), protein C (PC), and protein S (PS), factor V Leiden mutation, and prothrombin G20210A mutation, were excluded. Additional exclusion criteria for female candidates were pregnancy and breast feeding. At the day of blood donation the probands were additionally screened for infectious diseases, including testing for antibodies against HIV-1 and HIV-2, hepatitis C virus (HCV), Treponema pallidum, hepatitis B core antigen, and testing for hepatitis B surface antigen using chemiluminescent microparticle immunoassays (ARCHITECT i2000, Abbott Diagnostics, Wiesbaden, Germany). Additionally, HIV-1 and HCV was measured using in-house reverse transcriptase polymerase chain reaction methods [32,33].

### Materials

Human α-thrombin was obtained from CellSystems (St. Katharinen, Germany). Argatroban (Argatra^®^) was obtained from Mitsubishi Pharma (Düsseldorf, Germany). The 3’-biotinylated DNA-aptamer HD1-22 was synthesized and purified from Microsynth (Balgach, Switzerland). The fluorogenic peptide substrate I-1560 (Thrombin, Boc-Asp(OBzl)-Pro-Arg-AMC) was purchased from Bachem (Weil am Rhein, Germany). Alteplase (Actilyse^®^) was purchased from Boehringer Ingelheim (Biberach, Germany).

### Preparation and transfusion of autologous serum

Within 14 days prior to blood donation and transfusion of autologous serum, and until completion of post-transfusion blood sampling any antithrombotic or analgetic medication was prohibited. At the day of the donation 250 mL blood were drawn into blood pack units without anticoagulant (Fenwal, Lake Zurich, IL) that had been prefilled with rt-PA (Alteplase) to achieve a final concentration of 20 μg/mL. After incubation at RT for 2 h, blood bags were centrifugated (4 042 g, 20 min, 15°C) using a Roto Silenta RS cooling centrifuge (Hettich, Tuttlingen, Germany). After centrifugation another bag was connected using a sterile tubing welder (TSCD, Terumo, Tokyo, Japan) and the serum supernatant transferred to the new bag. Eventually the transfer line was disconnected with a sterile tube sealer (Composeal Mobilea, Fresenius Kabi, Bad Homburg, Germany). After repeating this procedure of centrifugation and transfer of the supernatant to a new bag, the serum preparation was frozen and stored at <-18°C until used. Sterility of serum preparations was tested using the BACTEC-FX sterility testing system (Becton Dickinson, Franklin Lakes, NJ).

At least four weeks after blood donation a single dose of 50 mL autologous serum was transfused intravenously at a rate of 100 mL/h under perfusor control using 18-gauge peripheral venous catheters (Braun Melsungen, Melsungen, Germany). Blood samples were drawn prior to transfusion and at intervals of 15, 30 (end of transfusion), 45 min, and 1, 3, 6, 24, and 48 h after the start of transfusion. A new venipuncture of an antecubital vein was performed at each sampling time point using 21-gauge winged infusion sets (Sarstedt, Nümbrecht, Germany). After discharge of the first 2 mL, blood was drawn into serum tubes, and citrate tubes (10.5 mM final concentration, Sarstedt, Nümbrecht, Germany). Citrate tubes contained argatroban (100 µmol/L final concentration) for thrombin measurement. Serum und plasma samples were obtained by centrifugation (2 600 x g, 10 min) within 30 min and stored at <-70°C.

### Measurement of free thrombin by OECA

The OECA for thrombin detection was performed in the microtiter plate format using white Maxisorp Fluoronunc microtiter modules (Nunc A/S, Roskilde, Denmark) as previously described [31]. Wells were initially coated with 10 μg/mL of bovine serum albumin (BSA)-biotin (100 μL/well) in coating buffer (30 mM Na_2_CO_2_, 200 mM NaHCO_3_, pH 9.0). After incubation at 4°C overnight, wells were washed by rinsing them three times with phosphate-buffered saline (PBS) washing buffer (PBS, 0.05% Tween 20, pH 7.4). Thereafter, wells were sequentially incubated at RT with PBS washing buffer containing 1 mg/mL BSA and 10 μg/mL streptavidin for 1 h, blocking buffer (PBS, 20 mg/mL BSA, 0.05% Tween 20, pH 7.4) for 2 h, and Tris (tris(hydroxymethyl)aminomethane)-buffered saline (TBS) washing buffer (TBS, 1 mmol/L each CaCl_2_ and MgCl_2_, 0.05% Tween 20, pH 7.6) containing 1 nmol/L 3’-biotinylated aptamers HD1-22 and 1 mg/mL BSA for 1h. After incubation, wells were washed with TBS washing buffer and samples added (100 μL/well). After incubation for 2 h at RT and subsequent washing with PBS washing buffer, the fluorogenic substrate I-1560 (100 μmol/L in washing buffer) was added and changes in fluorescence over time measured using a plate fluorescence reader (Synergy 2, BioTek Instruments, Bad Friedrichshall, Germany). Calibration curves covering a ½-log10 concentration range from 0 to 10 ng/mL thrombin (0–272 pmol/L) were processed in parallel. Data obtained from the calibrators were interpolated by 4-parameter curve fit and used to calculate the thrombin concentration in the samples. Samples and calibrators were assayed in triplicate.

### Measurement of F1+2

For the measurement of F1+2 the Enzygnost F1+2 (monoclonal) assay (Siemens Healthcare Diagnostics Products, Marburg, Germany) was applied. In this assay a monoclonal antibody directed against the carboxy-terminal sequence of F1+2 is used, that would also bind to prothrombin activation fragment F2. Therefore F1+2 in serum and plasma samples was subjected to adsorption with BaCl_2_. While F1+2 binds to the barium salts, F2 remains in the supernatant [2]. The adsorption was carried out by addition of BaCl_2_ to the samples to achieve a final concentration of 0.072 M BaCl_2_. Under agitation the mixtures were incubated for 30 min at RT and for 16–18 h at 4°C, and then centrifuged at 4°C for 30 min at 6 450 g to separate the insoluble barium salts from the supernatant. The Enzygnost F1+2 (monoclonal) assay was performed with the supernatant and the obtained results used to calculate the amount of F2 in the original serum and plasma samples. F1+2 concentrations were then calculated by subtracting the F2 concentrations from the measurement results obtained by the Enzygnost F1+2 (monoclonal) assay in the original serum and plasma samples.

### Measurement of other parameters

The STACLOT VIIa-rTF assay (Stago, Asnières sur Seine, France) was used to determine the concentration of FVIIa. Fibrinogen levels (Clauss method), and activity levels of factors (F) II, VII, XI, AT, plasminogen, α_2_-antiplasmin, and D-dimer were determined using an automated coagulation analyzer (BCS XP, Siemens Healthcare Diagnostics, Eschborn, Germany) and standard reagents (Multifibren U, Innovin, Actin FSL, Berichrom Antithrombin III, Berichrom Plasminogen, Berichrom α2-Antiplasmin Kit, INNOVANCE D-dimer). TAT concentrations were determined using the TAT micro assay (Siemens Healthcare Diagnostics Products, Marburg, Germany). Antigen concentrations of t-PA and PAI were determined using the TECHNOZYM t-PA Ag ELISA assay, and the TECHNOZYM PAI-1 Ag ELISA (Technoclone, Vienna, Austria). The PAP ELISA assay (DRG Instruments, Marburg, Germany) was used to quantify PAP.

### Data analysis

Elimination kinetics of D-dimer, F1+2, TAT, and PAP were analyzed using the PKSolver software [34]. The in vivo recovery (IVR) was calculated as the maximum absolute increase in plasma concentration after transfusion, divided by transfused dose per proband’s plasma volume. The probands’ plasma volume was calculated using the Sprenger-equations [35].

## Results

### Preparatory dose-finding experiments

In a first series of experiments serum was prepared as described using different concentrations of rt-PA in order to variate the concentration of generated D-dimer in the preparations. D-dimer and other hemostasis-related biomarkers were measured in a dilution series of these serum preparations in normal citrated plasma. By doing so, the in vivo peak plasma levels of these parameters after transfusion of the serum at different doses were estimated. The peak plasma levels of the biomarkers had to be high enough above the respective reference ranges, because continuously elevated levels during the period of blood draws after serum transfusion were necessary to assess the elimination kinetics. Using a final concentration of 20 μg/mL rt-PA in drawn whole blood, suitable levels of biomarkers were observed for a serum dilution of 1:50 and 1:100 in citrated plasma **(S1 Table)**. Assuming a plasma volume of 2 700 to 3 000 mL, these dilutions corresponded to transfusions of 27–60 mL serum in a proband. Therefore a volume of 50 mL serum was transfused in the in vivo experiments.

### Study population

16 healthy probands were eligible and agreed to join the study. One participant dropped out because he did not report to the study center for transfusion. The per-protocol group consisted of all subjects that completed transfusion of autologous serum and had measurements of all variables at all sampling time points. Only subjects of the per-protocol group were included in the statistical analysis. Fifteen volunteers, 10 of whom were female, with a mean age (range) of 29 (23–53) years completed the study. Their body mass index was 22.5 (19.6–27.5) kg/m^2^, body weight 68 (53–92) kg, and calculated plasma volume 2 980 (2 516–3 754) mL. Measurements results of hemostasis parameters in their serum preparations are shown in **Table 1**.

**Table 1:** Hemostasis Parameters in Transfused Serum Preparations.

Parameter	Reference range in plasma	Median (interquartile range)
D-dimer, mg/L	< 0.5	692 (497–1 311)
F1+2, nmol/L	< 0.34	881 (610–1 056)
TAT, ng/mL	0.1–3.9	147 330 (122 590–170 905)
PAP, ng/mL	163–606	317 036 (232 624–480 081)
Thrombin, ng/mL	nd	5.68 (4.61–8.22)
FVIIa, ng/mL	0.03–1.06	11.21 (9.37–12.04)
t-PA, ng/mL	< 10	38 920 (32 055–41 995)
PAI, ng/mL	4–43	456 (270–516)
Fibrinogen, mg/mL	1.80–3.55	nd
Antithrombin, %	85–120	64.5 (55.1–67.8)
Plasminogen, %	82–150	32.2 (28.8–43.8)
α_2_-antiplasmin, %	90–110	nd

For FVIIa n = 11, for all other parameters n = 15; nd, not detectable.

### Changes of hemostasis parameters through serum transfusion

**Fig 1:**
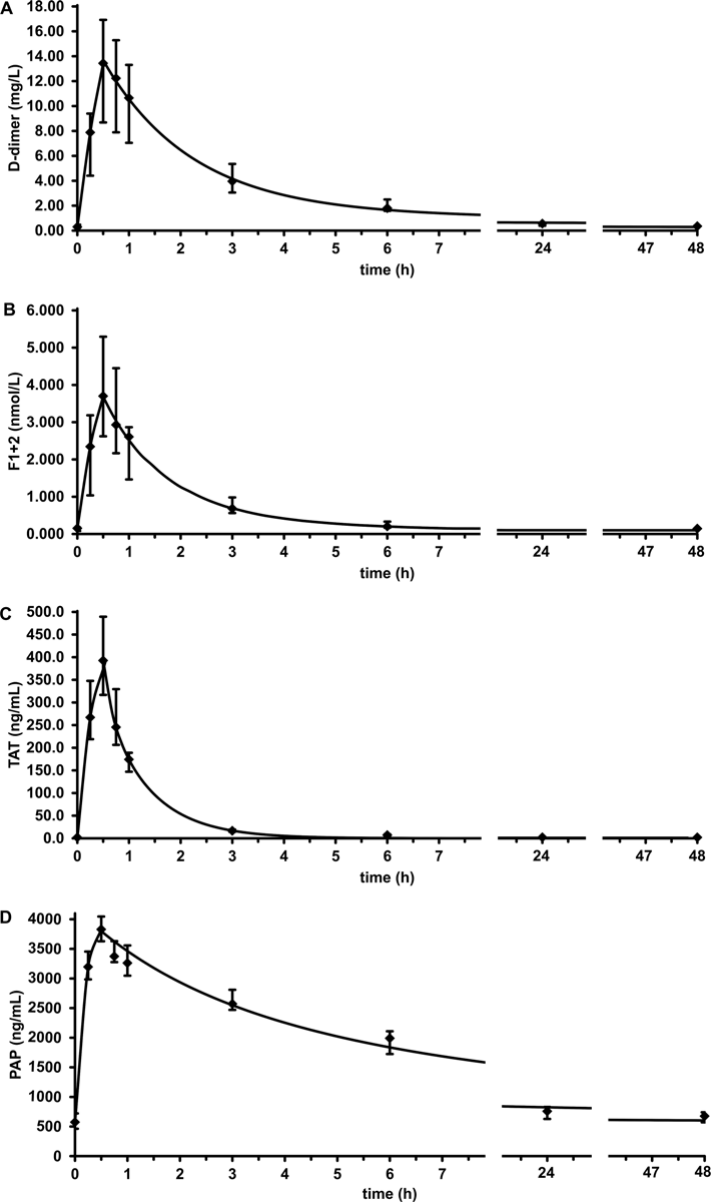
Plasma concentrations of (A) D-dimer, (B) F1+2, (C) TAT, and (D) PAP in healthy volunteers during and after autologous serum transfusion. Transfusion of 50 mL serum was started at t = 0 and completed at t = 0.5 h. Data points show the median of n = 15 probands, error bars show the interquartile range. Smooth curves show best least squares fit exponential decay functions.

Transfusions were well tolerated by all subjects and no adverse events of any kind were encountered during the study. Changes of hemostasis parameters from baseline to the end of serum transfusions in the probands’ plasma are shown in **Table 2**. Median (IQR) D-dimer levels increased from 0.30 (0.22–0.48) to 13.43 (8.69–16.93) mg/L, F1+2 levels from 0.15 (0.08–0.18) to 3.70 (2.62–5.29) nmol/L, TAT levels from 2.00 (2.00–2.19) to 393 (317–489) ng/mL, and PAP levels from 574 (462–721) to 3 829 (3 627–4 046) ng/mL. The rise of these four parameters were sustainable for the subsequent sampling time points **(Fig 1)**, allowing analysis of their elimination kinetics.

**Table 2:** Changes of Hemostasis Parameters Induced by Serum Transfusion.

Parameter	Baseline	t = 0.5 h	fold increase
D-dimer, mg/L	0.30 (0.22–0.48)	13.43 (8.69–16.93)	34.2 (24.9–78.6)
F1+2, nmol/L	0.15 (0.08–0.18)	3.70 (2.62–5.29)	28.9 (18.3–42.0)
TAT, ng/mL	2.00 (2.00–2.19)	393 (317–489)	189.6 (131.8–244.7)
PAP, ng/mL	574 (462–721)	3 829 (3 627–4 046)	7.0 (5.3–8.1)
Thrombin, ng/mL	nd	0.04 (nd—0.08)	NA
FVIIa, ng/mL	3.35 (2.29–3.79)	2.95 (2.15–4.05)	1.1 (0.9–1.2)
t-PA, ng/mL	1.96 (1.40–2.76)	26.08 (22.55–49.48)	17.4 (9.9–21.9)
PAI, ng/mL	8.85 (3.45–11.31)	4.51 (4.01–10.08)	1.1 (0.8–1.3)
Fibrinogen, mg/mL	2.75 (2.52–2.98)	2.55 (2.42–2.88)	1.0 (1.0–1.0)
Factor II, %	113 (103–129)	109 (101–130)	1.0 (0.9–1.0)
Factor VII, %	115 (103–131)	103 (101–126)	1.0 (1.0–1.0)
Factor XI, %	96 (92–100)	97 (89–106)	1.0 (1.0–1.0)
Antithrombin, %	100 (95–103)	96 (91–101)	1.0 (0.9–1.0)
Plasminogen, %	103 (96–137)	97 (94–126)	0.9 (0.9–1.0)
α_2_-antiplasmin, %	105 (98–115)	98 (89–105)	0.9 (0.9–0.9)

Data are presented as median (interquartile range) for n = 15 probands. nd, not detectable; NA, not applicaple.

With 0.044 (0.0–0.075) ng/mL, the median concentration of free thrombin was in a quantifiable range only in the plasma samples obtained at the end of serum transfusion. Similarly, t-PA levels were only elevated above the upper reference value at this sampling time point: Median t-PA increased from 1.96 (1.40–2.76) ng/mL at baseline to 26.08 (22.55–49.48) ng/mL at t = 0.5 h, but returned to levels below the upper reference value of 10 ng/mL with 6.08 (4.92–7.94) ng/mL measured at t = 0.75 h. All other measured hemostasis parameters, including FVIIa, did not increase through transfusion of the serum preparations. For all elevated parameters, maximum plasma levels were observed at t = 0.5 h.

### Analysis of elimination kinetics

The results of elimination kinetics analysis for D-dimer, F1+2, TAT, and PAP are presented in **Table 3**. Except for TAT measurements in two probands, and determinations of D-dimer and F1+2 in one proband each, a two-compartment model fitted the empirical individual subject data better than a one-compartment model.

**Table 3:** Elimination Kinetics of Hemostasis-related Biomarkers.

Parameter	D-dimer	F1+2	TAT	PAP
t_1/2_, h	15.8 (13.1–23.1)	1.9 (1.3–3.6)	0.7 (0.7–2.6)	10.8 (8.8–11.4)
IVR	0.88 (0.77–0.98)	0.26 (0.13–0.39)	0.15 (0.13–0.16)	0.64 (0.42–0.75)
AUC	58.6 (48.1–69.3)	6.6 (4.7–7.8)	401.0 (360.6–489.7)	37.6 (35.6–43.0)
CLR, mL/kg·h	7.2 (6.4–11.9)	64.3 (57.1–81.9)	269.7 (200.1–308.8)	6.2 (3.8–7.1)
MRT, h	15.4 (13.5–19.4)	2.4 (1.9–3.7)	0.9 (0.7–1.5)	15.2 (12.3–16.1)
Vd_ss_, mL/kg	167.9 (83.4–219.5)	272.0 (129.8–335.1)	242.5 (180.5–294.5)	78.0 (55.6–101.7)

Data are presented as median (interquartile range) for n = 15 probands. t_1/2_ indicates terminal half-life; AUC, area under the concentration-time curve; CLR, clearance; MRT, mean residence time; Vd_ss_, volume of distribution at steady state. Units for AUC are mg/L·h for D-dimer, nmol/L·h for F1+2, ng/mL·h for TAT, and μg/mL·h for PAP.

Of the four hemostasis-related biomarkers, the longest t_1/2_ was observed for D-dimer, with a median of 15.8 (13.1–23.1) h, followed by PAP with a median of 10.8 (8.8–11.4) h. Half-lives of F1+2 and TAT were markedly shorter, with a median of 1.9 (1.3–3.6) h and 0.7 (0.7–2.6) h, respectively. In one female subject outlying values were observed for the t_1/2_ of D-dimer (41.3 h), F1+2 (8.8 h), and PAP (23.4 h). With exclusion of this subject’s results the t_1/2_ of D-dimer in men was longer by a median of 4.2 h (95% CI, -9.0 to 17.4 h) versus women, of F1+2 by 0.8 h (CI, -3.5 to 1.8 h), and of PAP by 1.5 h (CI, -1.2 to 4.4 h). Although these differences of 29%, 47%, and 16% were not statistically significant, their extent was consistent with the differences in overall inter-individual variability of t_1/2_, which were higher for D-dimer and F1+2 than for PAP. Corresponding median gender-related differences in t_1/2_ of TAT were relatively minor (1.2 h; CI, -2.3 to 1.44 h), although TAT exhibited the second highest IQR of the four activation markers. Correlations between the half-lives of the four hemostasis-related biomarkers were statistically not significant.

Parameters for intravascular persistence were consistent with the estimations of terminal half-life, as a lower clearance and a longer MRT were calculated for D-dimer and PAP, than for F1+2 and TAT. The biomarker with the shortest half-life, TAT, also exhibited the highest clearance and the shortest MRT with a median clearance of 269.7 (200.1–308.8) mL/kg · h and MRT of 54 (42–90) min. The IVR of the biomarkers was also consistent with their terminal half-life, as the IVR was the highest for D-dimer with a median of 0.88 (0.77–0.98), followed by PAP with 0.64 (0.42–0.75), F1+2 with 0.26 (0.13–0.39), and TAT with 0.15 (0.13–0.16). With a median of 78.0 (55.6–101.7) mL/kg the VD_ss_ was markedly smaller for PAP than for D-dimer, F1+2, and TAT that demonstrated a median VD_ss_ of 167.9 (83.4–219.5) mL/kg, 272.0 (129.8–335.1) mL/kg, and 242.5 (180.5–294.5) mL/kg, respectively

## Discussion

Using autologous intravenous serum transfusions we simultaneously analyzed the in vivo elimination kinetics of the hemostasis-related biomarkers F1+2, TAT, PAP, and D-dimer.

To generate activation markers in a physiological matrix, whole blood was drawn into blood collection bags without anticoagulant but prefilled with the plasmin activator rt-PA in order to induce plasmin generation required for PAP and D-dimer formation. The serum prepared in this way contained levels of hemostasis-related biomarkers that were 500-fold to 7 000-fold higher than in plasma. It has been reported that serum transfusion induces thrombus formation in a mechanically occluded jugular vein segment in dogs [30]. This prothrombotic effect of serum has been attributed to the presence of active thrombin and other activated clotting factors in freshly prepared serum. We therefore measured the concentration of free thrombin using a highly sensitive OECA as recently reported from this laboratory in order to estimate the potential prothrombotic risk of serum transfusion [26]. A mean of approximately 6 ng/mL free thrombin was detected in the serum preparations, corresponding to a total amount of 300 ng or 0.9 U in an infusion of 50 mL [36].

The in vivo effects induced by infusion of thrombin were extensively studied in various animal models [37]. The results demonstrate that 0.9–1.6 U/kg/min over 2–5 hours were well tolerated without any signs of clotting activation or other side effects. In the present study the 50 mL of serum were infused over 30 min corresponding to 0.4 mU/kg/min in a human weighing 70 kg. As expected from these animal studies the serum transfusion was well tolerated in all probands and only 4% of the infused thrombin were detectable at the end of the serum transfusion. During the 48 h lasting observation period no quantifiable thrombin levels were detectable, demonstrating a rapid inactivation of infused thrombin by antithrombin in plasma in consistence with previous findings [38]. Moreover, the results of the thrombin measurements indicate that, if at all, only marginally activation of coagulation was induced by serum transfusion in this study, that might have induced de-novo generation of relevant amounts of F1+2, TAT, and D-dimer. The FVIIa levels in serum were only about threefold higher than in the plasma samples obtained at baseline. Thus, a total dose of 8 ng/kg FVIIa was infused in a human with 70 kg body weight in this study. In a previous study an about twofold increase of F1+2 plasma levels was observed after administration of 10 μg/kg recombinant FVIIa [39]. It is unlikely, that the more than 1 000-fold lower dose administered in our study induced a marked activation of coagulation. We also found no evidence, that the low amount of residual rt-PA significantly induced D-dimer formation in vivo. This finding is in line with a previous study showing no increase in plasma levels of D-dimer after infusion of low doses of rt-PA in healthy subjects [40].

Plasma levels of all four hemostasis-related biomarkers peaked at the end of the serum transfusion reaching concentrations that correspond to a strong activation of the hemostatic system. For example, D-dimer levels ranging from 8 to 16 mg/L can be measured in patients with severe thromboembolic complications or acute disseminated intravascular coagulation. The median fold increases of the four biomarkers between baseline and peak ranged from 7.0 for PAP to 189.6 for TAT. This high variation can most likely be explained by the inter-individual differences in baseline values before serum transfusion, the amount of the transfused biomarker, and the individual plasma volume. These determinants of the fold increase between baseline and peak are taken into account in the calculation of the IVR that showed results consistent with the other elimination kinetics parameters. It is unlikely that the differences in the fold increases were caused by endogenous biomarker formation, because an IVR <1.0 was observed for all four biomarkers.

The decay in plasma levels of all four activation markers followed a two-compartment model, in which part of the biomarker is distributed into a peripheral compartment. This is in line with previous studies on the pharmacokinetics of clotting factors such as factor IX and also supported by the clinical finding that increased levels of D-dimer can arise from extravascular D-dimer sources such as ascites [41]. With a median of about 16 h, the terminal half-life of D-dimer was nearly twice as long as the 9–10 h, which had been observed in previous human studies using radiolabeled D-dimer [23] or fibrinogen fragment D [21]. As the inter-individual variation of observed D-dimer half-lives was high in our study, the smaller number of healthy subjects in these studies of n = 3 [23] and n = 7 [21] might have led to different estimates. The calculated terminal half-life of 1.9 (1.3–3.6) h for F1+2 in our study was comparable with the findings of previous studies: In a study using radiolabeled F1+2 a half-life time of 1.5 h was calculated, based on measurements during the first 6 h after infusion [24]. When calculating pharmacokinetic parameters, an insufficient monitoring interval may result in underestimation of half-life [42]. This might explain the observed shorter half-life of F1+2 in comparison to our results, which were calculated based on measurements during 48 h. In another study the half-lives of F1+2 and TAT were calculated on the basis of plasma levels during cardiopulmonary bypass surgery and estimations of a computer model that accounted for the generation of thrombin, fibrin, marker clearance, hemodilution, blood loss, and transfusion [43]. With 88 ± 24 min the calculated half-life of F1+2 was nearly comparable with our results, but the estimated half-life time of TAT of 9 ± 6 min was substantially shorter than the 44 min (0.7 h) observed in our study [43]. However, the results obtained with radiolabeled TAT in animal studies (10–55 min) were in line with our findings [25–27]. One might speculate that the presence of high doses of unfractionated heparin in the cardiopulmonary bypass setting may have enhanced the elimination of TAT [44].

The observed half-life of PAP of 10.8 (8.8–11.4) h was also consistent with the results of a previous study in which a mean half-life of 0.52 days (12.48 h) was measured after infusion of radiolabeled α_2_-antiplasmin or plasmin and subsequent thrombolytic therapy [28]. In another study a mean half-life of 4.5 h was observed for PAP after administration of rt-PA in healthy human subjects, but blood samples were only drawn during an 4 h interval after infusion [40]. Therefore the half-life of PAP might have been underestimated in this previous study, as discussed above. Although the IVR of PAP was markedly below 1.0, it cannot be ruled out that the low dose of rt-PA in the transfused serum might have induced in vivo generation of plasmin, thereby causing a slower decay of PAP levels in plasma. However, t-PA levels in the probands’ plasma were elevated only during serum transfusion and returned to normal within 15 min thereafter, as expected from the reported half-life of rt-PA [45]. Therefore no significant amounts of PAP should have been generated after completion of serum transfusion, and any influence of a hypothetical rt-PA induced PAP generation on the later elimination phase of PAP and the calculation of its terminal half-life is unlikely.

**Fig 2:**
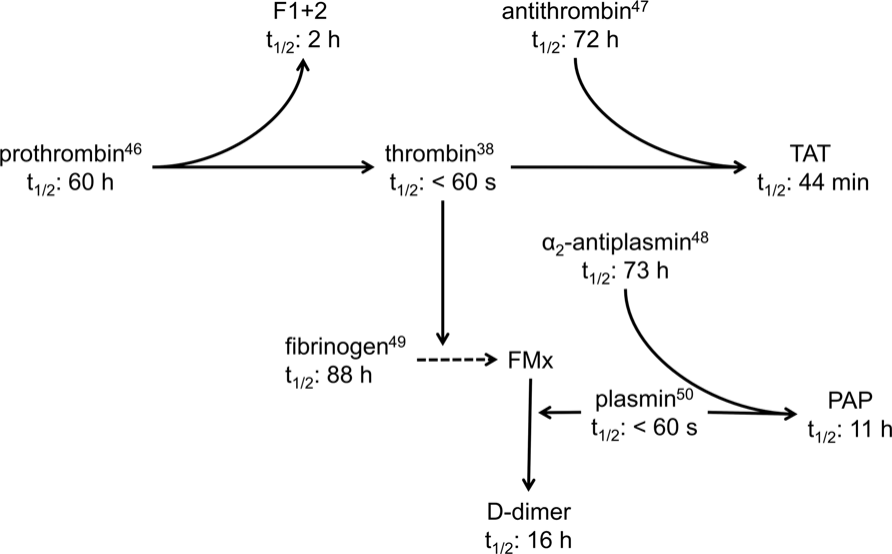
Half-lives of hemostasis-related biomarkers and their corresponding reactants. The relatively short half-lives of F1+2 and TAT qualify these biomarkers as dynamic biomarkers indicating changes in thrombin formation within hours, whereas D-dimer is a “long acting” biomarker indicating changes in fibrin degradation from day to day. The half-lives of the protease complexed serpins TAT and PAP differed markedly suggesting different elimination pathways. FMx indicates crosslinked fibrin.

The half-life of PAP is approximately 13-fold as long as that of TAT, although the half-lives of the serpins antithrombin and α_2_-antiplasmin and the active proteases thrombin and plasmin are nearly comparable **(Fig 2)** [38,46–50]. The clearance mechanisms of hemostasis-related biomarkers have not been identified so far. There is some evidence for the presence of a receptor-mediated hepatic clearance mechanism for TAT [51]. Differences in the elimination kinetics reported here, suggest that protease-serpin complexes are not cleared by a common pathway.

Our data demonstrate that the relatively short half-lives of F1+2 and TAT qualify these biomarkers to reflect changes in thrombin generation within hours whereas D-dimer is a long-acting thrombotic biomarker. The half-lives of the biomarkers summarized in **Fig 2**should be helpful to more accurately estimate the amount of thrombin and fibrin formation and fibrin degradation in various clinical states. Translating these results into clinical practice we come to the following conclusions: In patients presenting with increased levels of D-dimer simultaneously increased levels of F1+2/TAT and PAP are indicative for ongoing intravascular thrombin and fibrin formation. In contrast, F1+2/TAT levels within the reference range make continuous generation of thrombin unlikely. This especially applies for conditions where elevated D-dimer in the circulation might not be the result of ongoing coagulation and fibrinolysis but of distribution from the peripheral to the central compartment.

To sum up, the serum approach used in this study to assess the elimination kinetics of hemostasis-related activation markers is easy to perform and has proven to be safe and well tolerated. Therefore it should be extended to patients with impaired renal or hepatic function, patients with thrombophilia, or patients with antithrombotic medication, in order to assess different potential variables that might affect the elimination kinetics of D-dimer and other hemostasis-related biomarkers.

## Supporting Information

S1 Table Hemostasis Parameters in Serum Preparations. (DOCX)
