# Supplementary material for: Label-Free Kinetic Studies of Hemostasis-Related Biomarkers Including D-Dimer Using Autologous Serum Transfusion
Source: PLoS One. 2015 Dec 14;10(12):e0145012. doi: 10.1371/journal.pone.0145012 (PMC4684386; doi:10.1371/journal.pone.0145012)
Supplement: S1 Table — (DOCX) [file pone.0145012.s001.docx]

**S1 Table. Hemostasis Parameters in Serum Preparations.**

| **Parameter** | **reference range** | **undiluted** | **1 : 50 dilution*** | **1 : 100 dilution*** |
| --- | --- | --- | --- | --- |
| D-dimer, mg/L | < 0.5 | 338.0 ± 23.9 | 4.8 ± 2.2 | 2.9 ± 1.5 |
| F2/F1+2, nmol/L | ≤ 0.34 | 1 054.0 ± 65.9 | 19.8 ± 3.1 | 11.0 ± 4.3 |
| TAT, ng/mL | 0.1 - 3.9 | 154 098 ± 30 106 | 2 283 ± 917 | 1 340 ± 424 |
| PAP, ng/mL | 163 - 606 | 303 703 ± 26 720 | 5 298 ± 592 | 2 996 ± 223 |
| t-PA, ng/mL | ≤ 10 | 38 670 ± 976 | 638 ± 159 | 310 ± 51 |

Data are presented as mean ± standard deviation for n = 3 experiments. F2/F1+2 indicates the compound of prothrombin activation peptide F1+2 and F2; TAT, thrombin-antithrombin complex; PAP, plasmin-α_2_--antiplasmin complex; t-PA, tissue-type plasminogen activator. *Reference range and dilution refer to citrate anticoagulated plasma.
